# Can Paediatric Femoral Fracture Hip Spica Application be Done in the Outpatient Setting?

**DOI:** 10.5704/MOJ.2103.016

**Published:** 2021-03

**Authors:** ST Yap, NKL Lee, ML Ang, RW Chui, KBL Lim, M Arjandas, KPL Wong

**Affiliations:** 1 Department of Orthopaedic Surgery, KK Women's and Children's Hospital, Singapore; 2 Division of Surgery, KK Women's and Children's Hospital, Singapore

**Keywords:** children femoral fracture, hip spica casting, operating theatre, clinic plaster room, emergency department

## Abstract

**Introduction:**

Hip spica casting is a standard treatment for children with femur fractures. This study compares the outcomes of spica cast application, in terms of quality of fracture reduction and hospital charges when performed in operating theatre versus outpatient clinics at a local institution.

**Materials and Methods:**

A total of 93 paediatric patients, aged between 2 months to 8 years, who underwent spica casting for an isolated femur fracture between January 2008 and March 2019, were identified retrospectively. They were separated into inpatient or outpatient cohort based on the location of spica cast application. Five patients with metaphyseal fractures and four with un-displaced fractures were excluded. There were 13 and 71 patients in the outpatient and inpatient cohort respectively who underwent spica casting for their diaphyseal and displaced femur fractures. Variables between cohorts were compared.

**Results:**

There were no significant differences in gender, fracture pattern, and mechanism of injury between cohorts. Spica casting as inpatients delayed the time from assessment to casting (23.55 ± 29.67h vs. 6.75 ± 4.27h, p<0.05), increased average hospital stay (41.2 ± 31.1h vs. 19.2 ± 15.0h, p<0.05) and average hospital charges (US$1857.14 vs US$775.49, p<0.05). Excluding the un-displaced fractures, there were no significant differences in the period of cast immobilisation and median follow-up length. Both cohorts had a similar proportion of unacceptable reduction and revision casting rate.

**Conclusion:**

Both cohorts presented similar spica casting outcomes of fracture reduction and follow-up period. With spica cast application in operating theatre reporting higher hospital charges and prolonged hospital stay, the outpatient clinic should always be considered for hip spica application.

## Introduction

Femur fractures are the most common orthopaedic injury for which children require a hospital admission^1^. Treatment goals for these fractures include achieving fracture union while maintaining acceptable angulation and leg length, with the shortest inpatient stay and the least social and financial strain on the family^2^. Spica casting is the preferred treatment for children between six months and six years of age; other options include external fixation, compression plates and flexible intramedullary nails^3,4^. Immediate hip spica application in the emergency department (ED) has been described in numerous studies showing favourable results^4-6^. The advantages of immediate spica casting include a reduced length of hospital stay and lower hospital charges as compared to other treatment options^5,7-9^. Current literature has limited studies comparing spica casting in the operating theatre (OT) under general anaesthesia versus the ED or the plaster room (PR)^10^. This study aims to determine if the quality of fracture reduction is better in the OT to justify the higher hospitalisation charges in a high-volume tertiary children’s hospital. The higher hospitalisation costs must be justified by a higher quality of treatment. Studies have shown that the mortality rate increases when patients are discharged early due to bed shortages^11^. If the quality of treatment of spica casting is similar in the inpatient and outpatient settings, patients should be encouraged to visit the outpatient clinic to free up available bed vacancies in the inpatients for patients who need it more.

This study also aims to compare the economic burden on patients when they seek treatment in the inpatient versus the outpatient clinic. If the quality of treatment is similar in both inpatient and outpatient clinic, inpatient clinic with lower hospitalisation costs should be considered first to lessen the economic burden on the patients.

## Material and Method

This study is approved by the Institutional Review Board (Reference number: 2017/2654). A list of paediatric patients with femoral fractures who sought treatment at a single tertiary children’s hospital between 1 January 2008 to 31 March 2019 was generated using the diagnosis code. This study included patients between the age of two months to eight years who underwent hip spica cast application for their femur fractures. Patients with pathological fractures, open fractures, skeletal dysplasia and multiple traumatic injuries were excluded. A total of 93 patients were identified retrospectively and separated into the inpatient or outpatient group based on the facility where the spica cast application was performed. Inpatients were treated in the operating theatre (OT) while outpatients were treated in the emergency department (ED) or clinic plaster room (PR). In our institution, casts are applied in the ED with ketamine as a form of anaesthesia and in the OT with general anaesthesia. Of the 93 patients, 5 with metaphyseal fracture and 4 with un-displaced fractures were excluded from the study. These fractures were undisplaced with no issues of alignment or stability, hence, this group has been excluded as the outcomes would generally be excellent nonetheless. Overall, there were 13 patients in the outpatient cohort and 71 patients in the inpatient cohort, in which all underwent spica casting for their diaphyseal and displaced femur fractures. There were no criteria for determining which patients are treated in the outpatient and inpatient cohorts. It was strictly affected by the availability of resource and support in the Children's Emergency at the point of presentation. This is supported by the table showing that there is no statistical significance between the two different groups in terms of fracture characteristics Table I.

**Table I:** Baseline characteristics of inpatient and outpatient cohorts with displaced femoral shaft fractures

N = 84	Outpatient = 13	Inpatient = 71
Average Age	2.7 ± 2.55 years	2.9 ± 1.98 years
Girls	5	28
Boys	8	43
Left femur	9	43
Right femur	4	28
Completely displaced	13	71
Transverse fracture pattern	2	14
Oblique/spiral fracture pattern	11	57
High energy mechanism	5	38
Low energy mechanism	8	31
Unknown	0	2
Weight (kg)	11.2 ± 4.05	12.9 ± 4.41
Average initial fracture shortening	1.21 ± 1.31cm	1.26 ± 1.00cm
Average initial fracture varus/valgus angulation	18.0 ± 14.6°	14.9 ± 13.2°
Average initial fracture anterior-posterior angulation	19.0 ± 15.3°	16.9 ± 16.9°

In the outpatient cohort, once the fracture has been diagnosed by the orthopaedic surgical team, manipulation and reduction will be performed by the on-call orthopaedic surgeon and plaster technician. This would be done with image intensifier guidance in the children’s emergency under conscious sedation. The child will then be placed in a hip spica. The post-reduction angle measurements of the initial cast follow the same guidelines as the final radiographs.

In both groups, the families of patients received education on caring for patients with the cast to avoid potential cast complications. Patients would be referred to the general paediatric medical team and medical social worker (MSW) for evaluation of non-accidental injury (NAI) based on clinical suspicion, and they were discharged when appropriate. Fracture healing was monitored clinically and radiographically for all patients. After four to six weeks, the hip spica casts were removed if there was adequate fracture callus formation. If there was any concern of inadequate radiographic union, a removable above-knee backslab is applied for another four weeks. The clinical union was established when no tenderness was elicited on palpation of the fracture site with the cast removed. Patients were then permitted to bear weight and resume regular activities.

Each patient’s electronic and paper medical record was reviewed for his or her demographic data, admission details, femoral fracture characteristics and assessment during follow-up clinic visits. The duration of inpatient stay was calculated from the time of admission until the patient was deemed suitable for discharge from inpatient orthopaedic care. This was to account for the additional hospital stay while investigations for NAI were yet completed. The mechanism of injury was classified into low-energy for those resulting from a twisting injury or fall from standing height. Billing records were also obtained for each patient for the charges incurred during the period of inpatient stay.

Radiographic parameters used for an acceptable reduction of a paediatric femoral shaft fracture was referenced from Cassinelli *et al*^4^ who described hip spica casting in the emergency room. These measurements were performed on the last radiographs done during their follow-up visits for this fracture. During the final radiographs upon which the measurements were taken, the average duration of treatment was four to six weeks. For patients below two years of age, acceptable angulation was 30° in the sagittal and coronal planes, and 15mm of shortening. For patients two years of age and above, acceptable angulation was 15° in the coronal plane, 20° in the sagittal plane and 20mm of shortening. Alignment is taken as best possibly done in the hip spica cast but where the elastic nail is not indicated yet.

Statistical analysis was performed using chi-square tests to compare the variables between the two cohorts in a univariate sequence. Statistical significance was determined to be of p-value less than 0.05.

## Results

Both inpatient and outpatient cohorts had no significant differences in age, sex, laterality of injury, and the energy level of the mechanism of injury Table I. The average age of patients was 2.7 ± 2.55 years in the outpatient cohort and 2.9 ± 1.98 years in the inpatient cohort. The proportions of boys in the outpatient group were 61.5% (8 out of 13) and 60.6% (43 out of 71) in the inpatient cohort. 61.5% (8 out of 13) of the outpatient cohort suffered a high-energy mechanism while it was 53.5% (38 out of 71) in the inpatient cohort. The average weight of patients in the outpatient group was 11.2 ± 4.05kg and that in the inpatient group was 12.9 ± 4.41kg.

From our study, it was revealed that spica casting as an inpatient delayed the time from presentation to cast placement compared to an outpatient (23.55 ± 29.67h vs. 6.75 ± 4.27h). The inpatient cohort also reported lengthened the average hospital stay (41.2 ± 31.1h vs. 19.2 ± 15.0h) after correcting for two outpatients who had prolonged hospital stay due to MSW investigation and complication of pressure sore Table II. The average hospital charges of spica cast application in the inpatient cohort were about 1.5 times higher than that of the outpatient cohort (S$2549.19 vs S$1064.47; US$1857.14 vs US$775.49 [S$1:US$0.73, Nov 26 2018]).

**Fig. 1:**
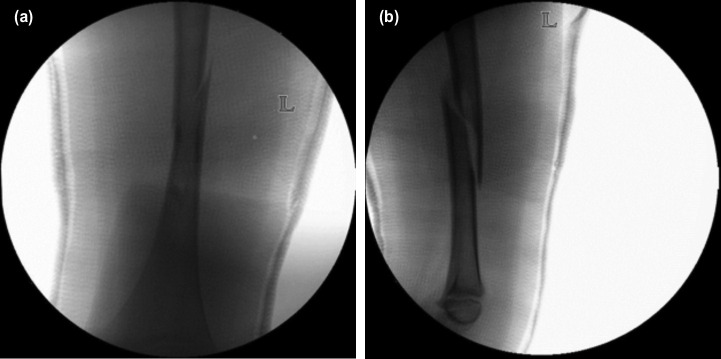
(a,b) Radiographs of the closed reduction and hip spica application of patient A.

**Fig. 2:**
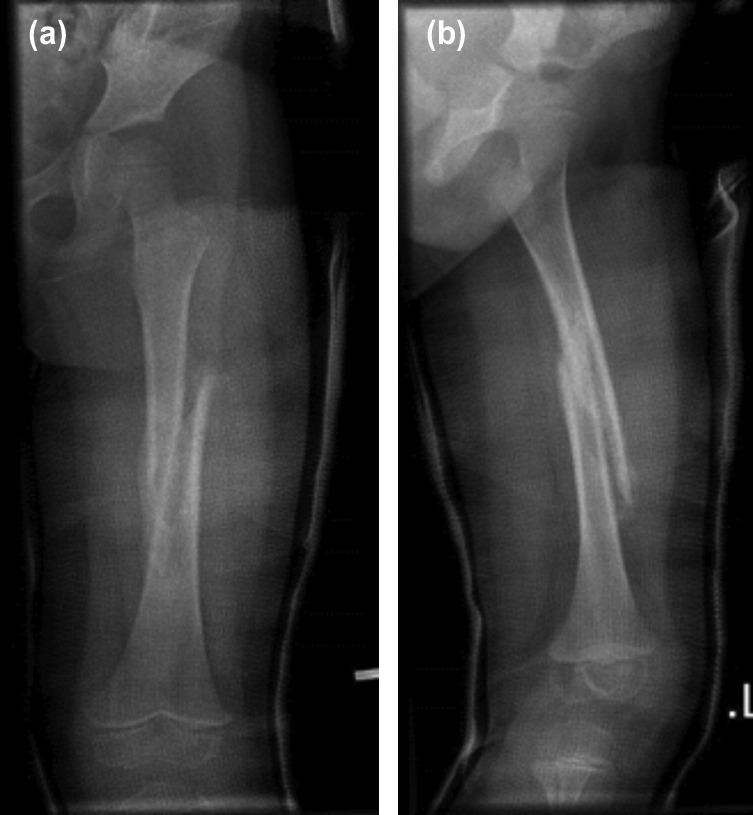
(a,b) Radiographs showing loosening of the initial cast around the thigh especially proximally after four weeks for patient A.

Two out of 71 (2.8%) in the inpatient cohort required recasting and 22.5% (n=16) received a removable above-knee cast after the hip spica was removed. This was compared to 7.7% (1 out of 13) and 15.3% (n=2) respectively in the outpatient cohort Table II. Most recasting were performed four weeks after the initial cast due to insufficient callus healing and because the cast is usually loose and dirty by the fourth week on follow-up (Fig. 1 and 2). Also, for suitable fractures, the cast was shortened to an above-knee cast instead of the hip spica. It was assessed by the surgeon in charge that the patients require further immobilisation. However, this is rare for both inpatient and outpatient cohorts as evident from the low percentages of patients requiring recasting. The outpatient and inpatient cohort had a comparable number of days of cast immobilisation (47 ± 12 days vs 50 ± 19 days), number of follow-up clinic visits (3.42 ± 1.62 days vs 3.83 ± 1.46 days), median number of days of clinic follow-up (75 days vs 116 days) and number of radiographs performed after spica casting (5.23 ± 1.64 vs 5.63 ± 1.43). There were no significant differences in those variables between the two cohorts Table II.

**Table II:** Outcome variables of displaced femoral shaft fractures between inpatient and outpatient cohorts

N = 84	Outpatient = 13	Inpatient = 71	p-value
Time ED* to spica (hours)	6.75 ± 4.27 h	23.55 ± 29.67 h	<0.05
Duration of stay (hours)	19.2 ± 15.0 h	41.2 ± 31.1 h	<0.05
Days in cast	47 ± 12 days	50 ± 19 days	0.469
Number of recasting spica	1	2	
Number of above-knee cast	2	16	
Costs of initial hospitalisation	US$775.49	US$1857.14	<0.05
Number of follow-up clinic visits	3.42 ± 1.62	3.83 ± 1.46	0.289
Median days of follow-up	75 days	116 days	0.365
Number of radiographs post spica	5.23 ± 1.64	5.63 ± 1.43	0.419
<2 years old	N=6	N=25	
Unacceptable final shortening (>15mm)	2	2	
Unacceptable varus/valgus (>30°)	0	0	
Unacceptable anterior-posterior (>30°)	0	1	
Unacceptable final alignment	2	3	
=>2 years old	N=7	N=46	0.928
Unacceptable final shortening (>20mm)	4	11	
Unacceptable varus/valgus (>15°) Unacceptable anterior-posterior (>20°)	3 3	8 11	
Unacceptable final alignment	5	25	

*ED: Emergency Department

In children of less than two years of age, the overall rate of unacceptable reduction was 33.3% (2 out of 6) and 12% (3 out of 25) in the outpatient and inpatient cohorts respectively. In the age group of 2 years and above, the overall rate was 71.4% (5 out of 7) and 54.3% (25 out of 46) respectively. The difference was not statistically significant.

## Discussion

This study is one of the few which compares the location of hip spica cast application, either in the outpatient clinic such as the ED and PR or the OT, for treatment of paediatric femoral fractures^5,10^. Mansour *et al*^10^ reported 100 patients being treated with immediate hip spica casting in either the OT or ED with similar outcomes with regards to the quality of fracture reduction and rates of complications. The number of patients in their outpatient cohort was much larger than the inpatient cohort and the spica cast application were performed by residents. Most of our patients had their spica cast application done as inpatients in the OT and performed by a consistent group of attending surgeons with the cast technicians assisted by residents. Numerous studies reported acceptable treatment outcomes with spica cast application in the OT but either did not have a comparison group or were comparing against other treatment methods^12-15^.

Our results demonstrate approximately twice the time to spica application, length of hospital stay, and initial hospitalisation bill was required for inpatients who underwent spica cast application in OT compared to those done in the ED or PR. This increase was comparable to a similar study reported by Mansour *et al*^10^. His team found a significant difference in the average time from ED admission to cast application between their cohorts (3.8h for outpatient and 11.5h for inpatient, P<0.0001). Duration of stay excluding NAI investigation was significantly longer for the inpatient cohort (30.5h vs. 16.9h, P=0.0002). Average hospital charges for spica casting in the inpatient cohort was 3 times higher when compared with the outpatient cohort (US$15,983 ± $3587 vs. US$5150 ± $1694, P<0.0001). Despite differences in geographical location and healthcare systems between both studies, these findings were consistent.

While the demographics of our study population was comparable in terms of age, gender, the proportion of high-energy mechanism to other studies, we found the time from ED admission to spica application in both the outpatient and inpatient cohorts was twice that reported by Mansour *et al*^10,4^. The average time from ED admission to spica application in our cohorts compared with that of their academic centre was 6.8h vs 3.8h in the outpatient group, and 23.6h vs 11.5h in the inpatient group respectively. We defined this time starting at the point the patient was registered in the ED and ending at the point of spica cast application. Immediate spica cast application has been described to be within 24, 48 and up to 72 hours by various authors and little studies are reporting the time from ED admission to spica cast application^5,16,17^. These differences are a result of multiple factors including the availability of location such as the OT, PR in the clinic and procedure room in the ED, availability of staff such as those involved in sedation/anaesthesia, monitoring and application of spica casts, availability of equipment such as the fluoroscopy machine^10^.

No significant differences in the care after the application of spica cast between the two cohorts were noted in terms of the number of days of cast immobilisation, follow-up period, and the number of follow-up radiographs performed. In a randomised controlled trial of spica cast application against external fixation, Wright *et al*^18^ reported the number of days of cast immobilisation to be 58 days. Siddiqui *et al*^19^ also reported the number of days to cast removal was six to eight weeks in their trial comparing immediate hip spica cast with skin traction followed by spica cast. The period of hip spica cast immobilisation was comparable amongst these studies involving hip spica casts.

**Fig. 3:**
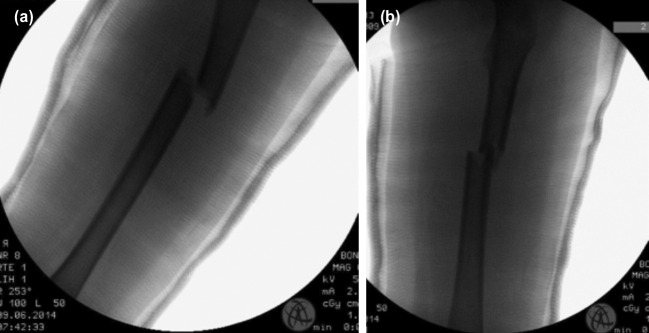
(a,b) Radiographs of the closed reduction and hip spica application of patient B.

**Fig. 4:**
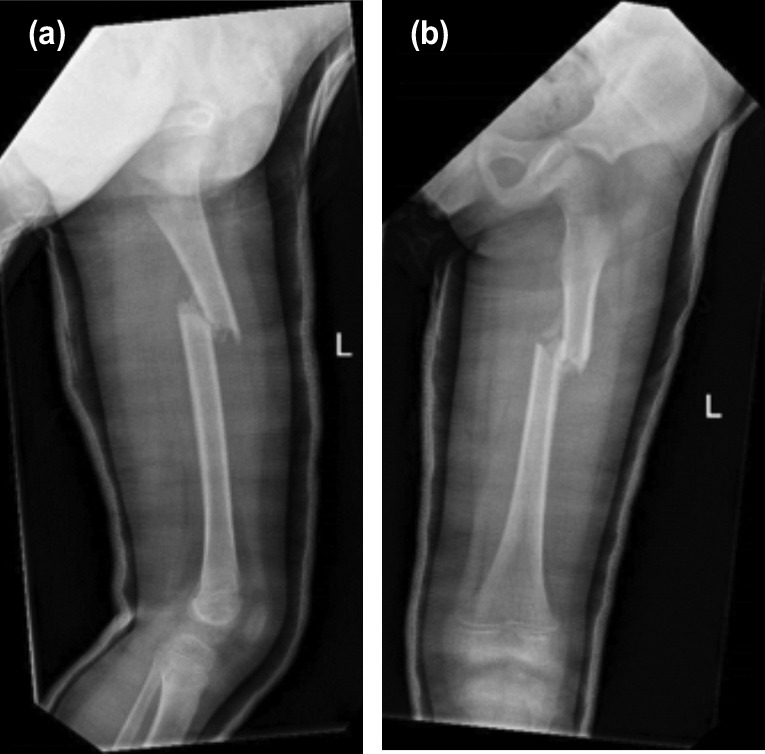
(a,b) Radiographs for the final shortening of patient B showing instability of the fracture.

Our proportion of unacceptable final alignment, which criteria was proposed by Cassinelli *et al*^4^, was comparable to that reported by other studies^20-21^. Current literature shows hip spica casting to have a malunion rate from 8.6% to 45%^15,20,24^. In a direct comparison with Mansour *et al’s*^10^ study for the age group of fewer than two years, their overall rate of unacceptable reduction was 11.1% (3 out of 27) and 40% (2 out of 5) in the outpatient and inpatient cohorts respectively, while we reported 33.3% (2 out of 6) and 12% (3 out of 25). In the age group of two years and above, they found an overall rate of unacceptable reduction of 30.8% (16 out of 52) and 31.3% (5 out of 16) in the outpatient and inpatient cohorts respectively, while we found it to be 71.4% (5 out of 7) and 54.3% (25 out of 46). Wright *et al*^18^ had found a similar overall rate of malunion to be 45% (25 out of 56) in a two-year follow-up in a randomised controlled trial comparing hip spica casting versus external fixation for paediatric femoral fractures. While our proportion of unacceptable alignment is 71.4% and 54.3% in the outpatient and inpatient cohorts respectively, it is worth noting that these children will eventually go on with good alignment because of their remodelling potential of femur fractures^25-26^. Our results also reflected that 30.8% (4 out of 13) of the outpatient cohort and 15.5% (11 out of 71) of the inpatient cohort had unacceptable final shortening of more than 20mm Table II. On average, further shortening from the initial is approximately 10 to 20mm. This final shortening would be due to the instability of the fracture despite the initial shortening of less than 20mm (Fig. 3 and 4). Hence, this shows the limits of the hip spica cast application.

In our institution, the number of hip spica casts performed in the outpatient clinics was much lesser than that performed in the OT. Firstly, ours is a high-volume ED and outpatient PR which tend to have limited capacity to support sedation for the procedure for extended periods. Secondly, existing work processes are well established for procedures to be performed in the OT where the sedation/anaesthesia team, fluoroscopy and casting equipment can be readily assembled. These reasons highlight the barriers to implementing a potentially cost-effective practice in our institution to perform hip spica casting out of the OT for femoral shaft fractures.

This study has several limitations. The retrospective nature of this study introduces the potential for selection bias. There were much more patients in the inpatient cohort (n=71) compared to the outpatient cohort (n=13) for the treatment of displaced femoral shaft fractures. However, the relative percentage difference was used for comparison in this study, hence, the disparity in cohort size has been taken into account during analysis. The small sample size in the outpatient cohort may not be able to detect statistically significant differences between the cohorts. Complications between the two treatment groups were not systemically documented and therefore not available for collection to be analysed in a meaningful manner. Clinical parameters such as pain scores, limb length and joint range of motion were not collected during follow-up. The cost of initial hospitalisation used in this study did not take into account investigations or charges not related to the care of affected femur fractures such as other radiographs for skeletal surveys, and subsequent procedures or outpatient charges. There was no long-term follow-up data available. These limitations may form the basis for further research with a potential for a prospective randomised controlled trial to develop an efficient and effective process for hip spica cast application in children with femoral shaft fractures.

## Conclusion

Paediatric hip spica casting in the inpatient and outpatient cohorts reported similar outcomes with regards to the quality of fracture reduction. With significantly higher hospital charges and longer hospital stay for spica application in the OT, this study supports outpatient clinics such as the ED and PR to be considered as alternative clinical areas for hip spica application in patients with femoral shaft fractures. Hip spica casts applied in the ED or PR is time efficient and cost-effective. Since both cohorts had a similar proportion of unacceptable reduction and revision casting rate, patients should be encouraged to opt for the outpatient clinic for treatment instead.
